# Molecular Phylogeny and Adaptive Mitochondrial DNA Evolution of Salmonids (Pisces: Salmonidae)

**DOI:** 10.3389/fgene.2022.903240

**Published:** 2022-06-17

**Authors:** Ying Wang, Fei Xiong, Zhaobin Song

**Affiliations:** ^1^ Hubei Engineering Research Center for Protection and Utilization of Special Biological Resources in the Hanjiang River Basin, College of Life Sciences, Jianghan University, Wuhan, China; ^2^ Sichuan Key Laboratory of Conservation Biology on Endangered Wildlife, College of Life Sciences, Sichuan University, Chengdu, China; ^3^ Key Laboratory of Bio-Resources and Eco-Environment of Ministry of Education, College of Life Sciences, Sichuan University, Chengdu, China

**Keywords:** Salmonidae, phylogenetic analyses, mitochondrial DNA, *dN/dS*, adaptive evolution

## Abstract

Salmonids are composed of anadromous and freshwater fishes, which is an important model for studying adaptive evolution. Herein, 49 salmonid complete mitochondrial genomes and those of two outgroups were used to infer a robust phylogeny for the family Salmonidae. The BI and RAxML phylogenetic trees based on 13 concatenated mitochondrial protein-coding genes showed well-supported nodes, and topologies were highly congruent. The concatenated 13 mitochondrial protein-coding genes, *ND2*, *ND3*, and *ND5* genes were shown to have significantly larger *dN/dS* ratios in anadromous species than in freshwater species of Salmonidae, but the *CYTB* gene had significantly smaller *dN/dS* in anadromous species. The FEL analysis identified positively selected sites and negatively selected sites in each mitochondrial protein-coding gene separately. The RELAX program revealed that the *ATP8* and *CYTB* genes supported intensified selection of the anadromous lineages. Our results demonstrated the phylogeny of Salmonidae and explored the mitochondrial DNA evolution pattern between anadromous and freshwater salmonids.

## Introduction

The family Salmonidae consists of three subfamilies with 11 genera and about 66 species: Coregoninae (whitefish, cisco, and inconnu), Thymallinae (grayling), and Salmoninae (lenok, huchen, salmon, trout, and char) (Gosline, 1963; Nelson, 2006). Salmonids have been intensively studied due to their high value in sport and commercial fisheries and their importance in a wide range of evolutionary and ecological questions of fishes (Groot and Margolis, 1991; Elliott, 1994; Hendry and Stearns, 2004). The most noteworthy feature of salmonids is that they could overcome the tremendous obstacles to return from distant oceans to natal streams for spawning. This notable migratory behavior of salmonids has raised many researchers’ attention to the evolution of migrations and the origin of this life history strategy (Mcdowall, 2001).

Mitochondria provide a great deal of energy *via* oxidative phosphorylation. Through comparison of the ratios of the rate of nonsynonymous nucleotide substitution to that of synonymous substitution (*dN/dS*), natural selection acting on protein-coding genes was ever characterized (Nei, 2005). A negative correlation was found between the *dN/dS* ratio and locomotive speed in birds and was also found for the *dN/dS* ratio and locomotive speed by examining 214 complete mammalian mitogenomes (Shen et al., 2009). Similarly, the teleost migratory ability has been reflected by selection patterns observed in mitochondrial genomes (Sun et al., 2011). Most species of Salmonidae are distributed throughout the Northern Hemisphere, including freshwater and anadromous species (Nelson, 2006). In particular, within Salmonidae, whitefish in the genus *Prosopium*, grayling (subfamily Thymallinae), lenok (*Brachmystax* spp.), and huchen/taimen (*Hucho* spp.) are exclusively freshwater species, while other salmonid groups are anadromous (Myers, 1949; McDowall, 1988; Davidson, 2013). Therefore, the *dN/dS* ratios of salmonid mitochondrial genomes could be estimated to test whether the mitochondrial protein-coding genes of the anadromous and freshwater salmonids have undergone different selective constraints.

Molecular systematics and phylogenetic investigations of salmonids have been elaborated in the previous studies (Crespi and Fulton, 2004; Phillips et al., 2004; Wilson and Turner, 2009; Shedko et al., 2012; Shedko et al., 2013; Macqueen and Johnston, 2014). Crespi and Fulton (2004) addressed the phylogeny of salmonid fishes based on 269 GenBank sequences of mitochondrial DNA and nuclear DNA, but they could not resolve the phylogenetic relationships including the genus-level and species-level relationships substantially since the freshwater species, *Hucho bleekeri*, *Hucho taimen*, and *Brachymystax lenok tsinlingensis* were not included. The recent study focused on elucidating the systematic relationships among salmonid fishes, but the phylogenetic tree topology was labeled with low support values (Crête-Lafrenière et al., 2012). Macqueen and Johnston (2014) combined protein (7222 AA), or nucleotide of truly orthologous nuclear genes and protein data (3790 AA) of mitogenome to conduct a phylogenetic analysis to reveal whole-genome duplication is decoupling from species diversification. This study recovered a Thymallinae–Coregoninae sister relationship with maximal support. Jacobsen et al. (2015) utilized 107 full mitogenomes of recently diverged species and lineages of whitefish to search for signals of positive selection at the mitogenome level. However, the evolution acting at the mitogenome level between freshwater and anadromous salmonids was unknown so far.

In the present study, 49 salmonid complete mitochondrial genomes, as well as those of two outgroups, were chosen to reconstruct the phylogeny for the family Salmonidae. At the same time, we investigated the potential selective pressure changes at the mitochondrial genome level between freshwater and anadromous salmonids. The purpose was to address the phylogenetic relationships for the family Salmonidae and explore the adaptive evolution patterns between freshwater and anadromous salmonids.

## Materials and Methods

### Data Collection and Analysis

All available complete mitogenomes of salmonids (*n* = 49) plus two outgroups *Esox lucius* (NC_004593) and *Osmerus mordax* (NC_015246) were downloaded from the National Center for Biotechnology Information (http://www.ncbi.nih.gov/). The corresponding accession numbers for the newly determined sequences and previously published sequences are listed in Supplementary Table S1. The nucleotide sequences of the 13 protein-coding genes (PCGs) were first extracted using purpose-built Perl scripts based on annotations and then separately aligned according to their corresponding amino acid translations by the software application TranslatorX (Abascal et al., 2010). The concatenated nucleotide sequence alignment from 13 PCGs (total = 11,355 bp) without stop codons was generated with our in-house scripts to conduct the phylogenetic analysis. Prior to the phylogenetic reconstruction, the extent of substitution saturation was estimated separately for entire codons and for the first, second, and third codon positions of the concatenated alignment using DAMBE 6.4.107 (Xia, 2017). The pairwise nucleotide diﬀerences (transitions and transversions) were plotted against the GTR genetic distance (Supplementary Figure S1).

### Phylogenetic Analysis

To improve the reliability of the phylogenetic analysis, the best-fit partitioning scheme across each gene and codon position was determined for each data set under the Bayesian information criterion by PartitionFinder software (Lanfear et al., 2012). Both partitioned maximum likelihood (ML) and Bayesian Inference (BI) approaches with the selected partition scheme were employed to reconstruct the phylogenetic relationships among salmonids. RAxML 7.0.3 (Stamatakis, 2006) with 1,000 nonparametric bootstrap replicates and MrBayes 3.1.2 (Ronquist and Huelsenbeck, 2003) were used to construct maximum likelihood (ML) and Bayesian inference (BI) trees, respectively. The ML trees were inferred using RAxML with the GTRGAMMA model. The Bayesian posterior probabilities were estimated using the Markov Chain Monte Carlo (MCMC) method with one cold chain and three heated chains for 20,000,000 generations, with every 1000th sample being retained. Twenty-five percent of the samples (5000 samples) from the burn-in were discarded, and the resulting trees were used to generate a 50% majority consensus tree with posterior probabilities. All MCMC runs were repeated twice to confirm a consistent approximation of the posterior parameter distributions.

### Analyses of Selection

Based on the aforementioned optimized tree topology from the concatenated 13 protein-coding genes, the ratio of nonsynonymous (*dN*) to synonymous (*dS*) substitutions rates (*dN/dS*) for concatenated gene sequences and each of the 13 mitochondrial protein-coding genes were computed separately to provide an indication of a change in selective pressure (Yang, 2007). The CODEML program of the PAML4.4 package (Yang, 2007) was implemented to assess whether the mitochondrial protein-coding genes of salmonids have undergone different selective constraints among freshwater and anadromous salmonids. Herein, model 1 with a free ω (*dN/dS*) ratio was estimated separately for each branch of the robust tree topology. The Wilcoxon rank-sum test was assigned to compute the level of statistical significance. All the statistical analysis was performed with SPSS 13.0 statistical package (SPSS Inc., Chicago, IL). To explore the difference in selective constraints between freshwater and anadromous salmonids, we applied the branch-site test from BUSTED (branch-site unrestricted statistical test for episodic diversification) (Murrell et al., 2015) to identify the branches under positive selection, which was implemented in the HyPhy package (Pond et al., 2005). Additionally, the fixed effects likelihood (FEL) approach in HyPhy software, which is more powerful than the CODEML program for detecting the individual sites subjected to episodic diversifying selection, was used to detect site-specific selection pressure. The FEL approach was run using the best-fitting nucleotide substitution model for each gene that was identified by jModelTest 2.1.3 (Darriba et al., 2012) on the ML phylogenetic tree. The subtree consisting of the 31 anadromous salmonids was specified as the foreground branch and tested, while the rest of the branches shared an arbitrary *dN/dS* ratio. Two models were nested in this method: *H0*, *dN* = *dS* (the neutral model), and *HA*, where *dN* and *dS* are estimated independently (the selection model). When the LRT is significant, if *dN* > *dS*, the site is declared to be under positive selection, otherwise the site is under negative selection. A nominal significance level of 0.1 for the likelihood ratio test was chosen based on the desired power of this analysis. RELAX is most useful for identifying trends and/or shifts in the stringency of natural selection on a given gene (Wertheim et al., 2015). Therefore, we used the program RELAX in HyPhy to distinguish intensified selection from the relaxed selection using 31 anadromous salmonids as the test lineages. RELAX then was used to test for the relaxed/intensified selection by introducing the parameter k. A significant result of k > 1 would indicate an intensified selection on test lineages, and a significant result of k < 1 would indicate a relaxed selection on test lineages.

## Results

### Characteristics of Salmonid Mitochondrial Genomes

The mitochondrial genome sizes of salmonids ranged from 16,526 to 16,997 bp. The mitochondrial genome contains 13 protein-coding genes, two ribosomal RNA genes, 22 transfer RNA genes, and a putative control region (CR) which is similar to those of other teleosts (Broughton et al., 2001; Saitoh et al., 2006). The length variations resulted from the control regions. The concatenated data set consisted of 11,355 bp from 13 PCGs of 51 mitochondrial genomes. There were 5,058 variable sites and 4,153 parsimony informative sites among the identified sites. The saturation test of the concatenated alignments was derived using DAMBE 6.4.107. There was no saturation for any codon position in the concatenated alignments (Supplementary Figure S1). The best-fit partitioning scheme across each gene and codon position is determined and shown in Table 1.

**TABLE 1:** Partition scheme of 13 PCGs used in this study.

Subset for 13 PCGs	Best model	Partition scheme
1	GTR+I+G	ND4_1st, ND5_1st, ATP6_1st, ATP8_3rd, ND1_1st, ND2_1st, and ND3_1st
2	HKY+I	ATP6_2nd, COX1_2nd, COX2_2nd, COX3_2nd, CYTB_2nd, and ND1_2nd
3	GTR+I+G	ND4_3rd, ND5_3rd, ATP6_3rd, CYTB_3rd, ND1_3rd, ND2_3rd, and ND3_3rd
4	SYM+I+G	ND4L_1st, ATP8_1st, COX1_1st, COX2_1st, COX3_1st, and CYTB_1st
5	GTR+I+G	ND4_2nd, ND4L_2nd, ND5_2nd, ND6_2nd, ATP8_2nd, ND2_2nd, and ND3_2nd
6	GTR+I+G	ND4L_3rd, COX1_3rd, COX2_3rd, and COX3_3rd
7	GTR+G	ND6_1st
8	GTR+G	ND6_3rd

### Phylogenetic Analysis From Mitochondrial Genes

**FIGURE 1:**
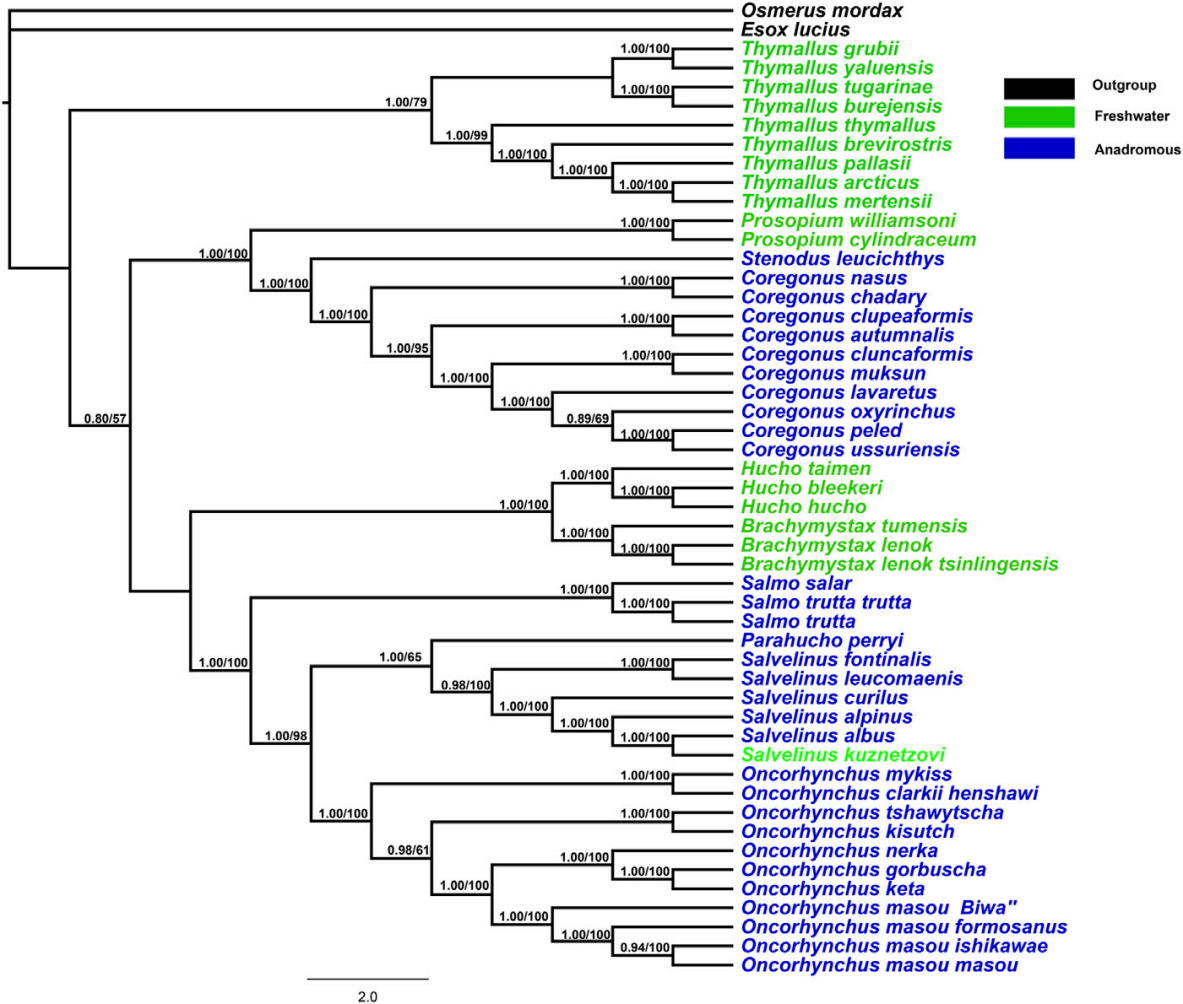
Phylogenetic tree constructed for combining 13 mitochondrial protein-coding gene orthologs using RAxML and Bayesian inference methods. MrBayes with GTR+I+G model was used. The Bayesian posterior probabilities and bootstrap support values are indicated at the nodes.

The BI and ML trees obtained from 13 mitochondrial protein-coding genes (total = 11,355 bp) revealed a consistent topology with high bootstrap values and posterior probabilities (Figure 1). Both freshwater and anadromous salmonids were mapped onto the phylogenetic topology. The phylogenetic results indicated that anadromous salmonids clustered as one monophyletic group in Coregoninae and clustered as a polyphyletic group in Salmoninae. Within Salmonidae, the freshwater Thymallinae emerged as a sister group to all other salmonids with high support values (0.80/100). There was a high support value for a sister relationship between both Coregoninae and Salmoninae subfamilies. Within the subfamily Coregoninae, three genera *Coregonus*, *Prosopium*, and *Stenodus* exhibited the evolutionary relationships [*Prosopium* (*Coregonus*, *Stenodus*)]. The phylogenetic analysis of the data sets produced six distinct and well-supported evolutionary groups for the subfamily Salmoninae as follows: *Brachymystax*, *Hucho*, *Salmo*, *Parahucho*, *Salvelinus*, and *Oncorhynchus*, which were supported by high support values. *Brachymystax* and *Hucho* were retained with strong nodal support (1.00/100) in the basal position of the trees orderly followed by the genera *Salmo*, *Parahucho*, *Salvelinus*, and *Oncorhynchus*. When we plotted the behaviors on the phylogeny of salmonids, we found there were three transitions from freshwater salmonids to anadromous salmonids (Figure 1). The results suggested the migratory behaviors of salmonids resulted from the multiple evolutionary origins.

### Analyses of Selection on Mitochondrial DNA

**FIGURE 2:**
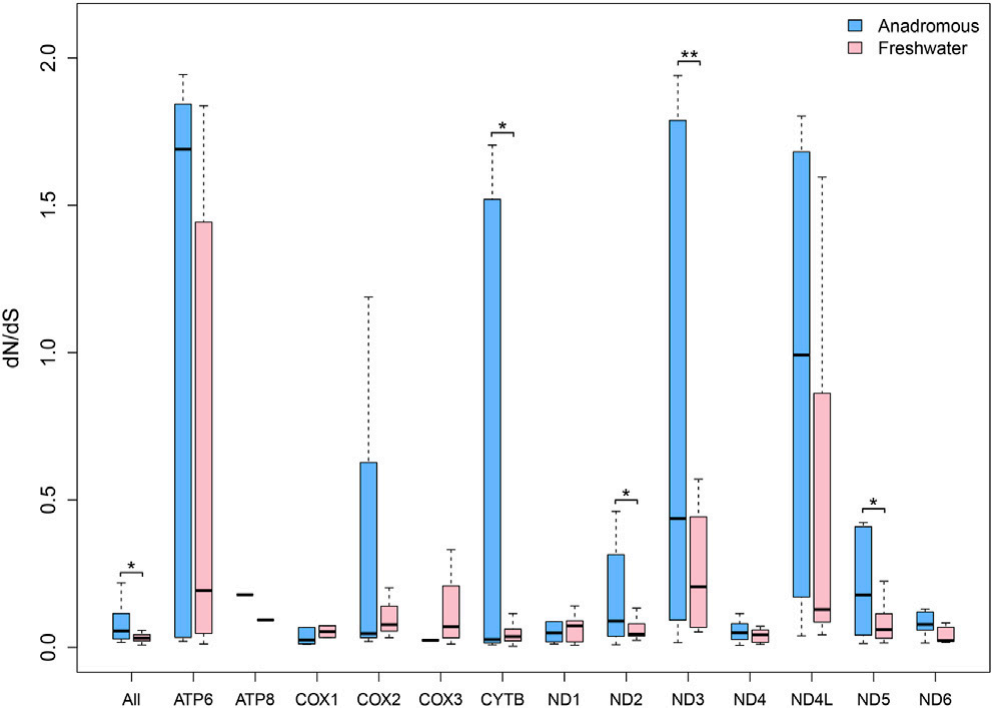
*dN/dS* ratios for each of 13 mitochondrial protein-coding genes of freshwater and anadromous salmonids.

The salmonids can be grouped into two categories: anadromous salmonids (marine\freshwater) and freshwater salmonids. The freshwater salmonids comprised 18 species, while the remaining 31 species are anadromous. We found that the overall *dN/dS* ratio for the concatenated 13 protein-encoding genes of the anadromous group was significantly larger than that of the freshwater group. Also, with respect to each gene separately, most mitochondrial genes of the anadromous group had larger *dN/dS* ratios than that of the freshwater group (Supplementary Table S2). Especially, *ND2*, *ND3*, and *ND5* genes possessed significantly larger *dN/dS* ratios in the anadromous group than that in the freshwater group (Figure 2). But the *CYTB* gene had significantly smaller dN/dS in the anadromous group.

When 31 anadromous salmonids were specified as the foreground branch, the FEL analysis identified positively selected sites and negatively selected sites in each mitochondrial protein-coding gene separately (Table 2). The RELAX program was used to compare the selective pressures on different parts of the tree, and the *ATP8* and *CYTB* genes supported intensified selection on the anadromous lineages among the 13 mitochondrial protein-coding genes (Table 3). BUSTED provided evidence that at least one site on at least one test branch has experienced diversifying selection. The results showed that the *ND5* gene evolved under positive selection in freshwater lineages, while the *ND5* gene and *ND6* gene evolved under positive selection in anadromous lineages. Therefore, it is reasonable to assume that the *ND6* gene might play an important role in the evolution of the migratory behavior of salmonids.

**TABLE 2:** Statistics of positively selected sites and negatively selected sites derived from the FEL (fixed effects likelihood) method.

Gene name	Number of positively selected sites	Number of negatively selected sites
ATP6	0	26
ATP8	0	2
COX1	0	91
COX2	0	37
COX3	0	71
CYTB	0	148
ND1	0	103
ND2	1	83
ND3	1	27
ND4	0	140
ND4L	1	2
ND5	0	148
ND6	0	53

**TABLE 3:** Intensified and relaxed selection for mitochondrial protein-coding genes on the anadromous branches.

Gene name	Selection intensification (K-value)	*p*-value	LR (likelihood ratio)
*ATP6*	K = 1.22	*p* = 0.261	LR = 1.26
*ATP8*	K = 40.93	*p* = 0.013	LR = 6.15
*COX1*	K = 0.98	*p* = 0.882	LR = 0.02
*COX2*	K = 0.93	*p* = 0.412	LR = 0.67
*COX3*	K = 1.13	*p* = 0.717	LR = 0.13
*CYTB*	K = 1.32	*p* = 0.012	LR = 6.38
*ND1*	K = 0.99	*p* = 1.000	LR = −0.00
*ND2*	K = 0.94	*p* = 0.632	LR = 0.23
*ND3*	K = 0.98	*p* = 0.859	LR = 0.03
*ND4*	K = 0.88	*p* = 0.170	LR = 1.88
*ND4L*	K = 1.41	*p* = 0.182	LR = 1.78
*ND5*	K = 0.94	*p* = 0.499	LR = 0.46
*ND6*	K = 0.89	*p* = 0.394	LR = 0.73

Note: a significant K > 1 would indicate intensified selection on test lineages, and a significant K < 1 would indicate relaxed selection on test lineages. The mitochondrial gene with *p* values less than 0.05 are shown in red.

## Discussion

The phylogenetic trees based on 13 concatenated protein-coding genes showed well-supported evolutionary relationships of the Salmonidae family, covering more genera (*n* = 10), compared to the previous salmonid mitogenome studies (Yasuike et al., 2010; Wang et al., 2011; Jacobsen et al., 2012; Campbell et al., 2013). Our present analyses yielded strong support for the basal position of Thymallinae, followed by a sister relationship between Coregoninae and Salmoninae, which is congruent with the recent molecular phylogenetic analyses based on whole mitochondrial genome sequences at the subfamily level (Li et al., 2010; Campbell et al., 2013; Macqueen and Johnston, 2014). However, the previous studies have revealed the inconsistency of the subfamily relationships that Coregoninae is in an ancestral phylogenetic position within Salmonidae and the sister relationship of Thymallinae and Salmoninae using the few mitochondrial genomes or a single nuclear locus (Yasuike et al., 2010; Wang et al., 2011; Shedko et al., 2012). The inconsistency is mainly resulting from the inclusion of only one *Coregonus* genus in phylogenetic analyses. Additionally, the phylogenetic analysis based on 78 nuclear gene sets suggested that the subfamily Thymallinae is in an ancestral phylogenetic position within Salmonidae, and the Coregoninae is the sister group to Salmoninae (Koop et al., 2008). Thymallinae and Salmoninae were clustered as a clade that is the sister group to the Coregoninae in the morphologically-based analysis of salmonid phylogenetic relationships (Wilson and Williams, 2010). The disagreements in subfamily relationships within the family Salmonidae that occurred in the previous studies might have resulted from the lack of the representative genera, the choice of outgroups, and different methodologies. In addition, the characteristics of mitochondria including linkage, absence of recombination, and small effective populations might limit their applicability in phylogenetic reconstruction (Birky et al., 1989; Tanya and Kumar, 2010). The evolutionary relationship is {[*Thymallus*, (*Prosopium*, (*Coregonus*, *Stenodus*))], [(*Hucho*, *Brachymystax*), (*Salmo*, (*Oncorhynchus*, (*Parahucho*, *Salvelinus*)))]} among these genera. Within the subfamily Coregoninae, the genus *Prosopium* occupied a basal position followed by a sister clade of *Coregonus* and *Stenodus*, which is in agreement with the early phylogenetic studies (Stearley and Smith, 1993; Macqueen and Johnston, 2014). The subfamily Thymallinae includes a single genus *Thymall*, which is generally accepted (Nelson, 2006; Yasuike et al., 2010). An extended dataset of salmonid mitochondrial genomes provided good resolution for the phylogeny of the family Salmonidae. Although some limitations occurred in mtDNA-based phylogeny, this study would keep the door open for subsequent work to address with additional datasets.

With respect to the subfamily Salmoninae, the evolutionary relationship based on the mitochondrial genes was consistent with the previous studies (Crespi and Fulton, 2004; Phillips et al., 2004; Yasuike et al., 2010). The results supported a sister relationship between the genus *Salvelinus* and *Oncorhynchus*, instead of *Salmo* and *Oncorhynchus* as expected from the morphology and ribosomal *ITS1* sequences (Phillips and Oakley, 1997), which agreed with the suggestions by Oakley and Phillips (1999) and Koop et al. (2008). The valid genus of the family Salmonidae was modified as *Coregonus*, *Stenodus*, *Prosopium*, *Thymallus*, *Hucho*, *Brachymystax*, *Salmo*, *Parahucho*, *Salvelinus*, and *Oncorhynchus*. As reported in the previous studies, the genera *Prosopium*, *Thymallus*, and *Hucho* are strictly freshwater species while other genera are anadromous species (Davidson, 2013). However, the freshwater and anadromous species were not clustered as monophyly on the phylogenetic trees, respectively, which reflected that anadromous species evolved at least twice from the freshwater salmonid ancestors (in Coregoninae and Salmoninae, respectively).

Sun et al. (2011) concluded the mitochondrial protein-coding genes of migratory fishes accumulate fewer nonsynonymous mutations than nonmigratory fishes. It suggested that the functional constraints act on mitochondria due to energy metabolism and influences the evolution of mitochondrial-encoded proteins in teleosts. At the same time, the analysis results suggested that *CYTB*, *ND1*, *ND3*, *ND5*, and *COX3* genes may have more important roles in energy production. Garvin et al. (2011) examined the codon sites of 12 protein-coding genes to identify what role natural selection may have played in the divergence of the mitochondrial-coding proteins among salmonids. Therefore, we investigated the adaptive evolution of salmonids and explored the selective patterns of freshwater and anadromous salmonids from mitogenomic perspectives. The concatenated 13 mitochondrial protein-coding genes were shown to have significantly larger *dN/dS* in the anadromous species than in the freshwater species of Salmonidae. Moreover, the *ND2*, *ND3*, and *ND5* genes were shown to have significantly larger *dN/dS* in the anadromous species than in the freshwater species of Salmonidae. But the *CYTB* gene had significantly smaller *dN/dS* in the anadromous fishes. This result is inconsistent with the conclusion drawn by 401 teleost complete mitochondrial genomes in the previous study (Sun et al., 2011). This inconsistency might result from the complex migratory behavior of salmonids or the small sample size (*n* = 51) available for this test in this study. Taken together, our results demonstrated that anadromous salmonids accumulated more nonsynonymous mutations in the mtDNA than freshwater salmonids. The result means migratory ability has significant effects on the mitochondrial *dN/dS* variation, which is reflected by the mitochondrial-encoded gene evolution. In conclusion, the adaptive mtDNA evolution pattern associated with complex migratory behavior was reflected in the specific group of salmonids. Through the branch-site model in HyPhy software, we detected the positively selected genes and sites, which suggested that mitochondrial protein-coding genes evolved at the molecular level to cope with the complex anadromous behaviors.

## Data Availability

The datasets presented in this study can be found in online repositories. The names of the repository/repositories and accession number(s) can be found in the article/Supplementary Material.

## Supplementary Material

Supplementary Figure S1 Saturation tests for any codon position in the concatenated alignment.
