# Supplementary figures and images for: Molecular Phylogeny and Adaptive Mitochondrial DNA Evolution of Salmonids (Pisces: Salmonidae)
Source: Front Genet. 2022 Jun 17;13:903240. doi: 10.3389/fgene.2022.903240 (PMC9249015; doi:10.3389/fgene.2022.903240)

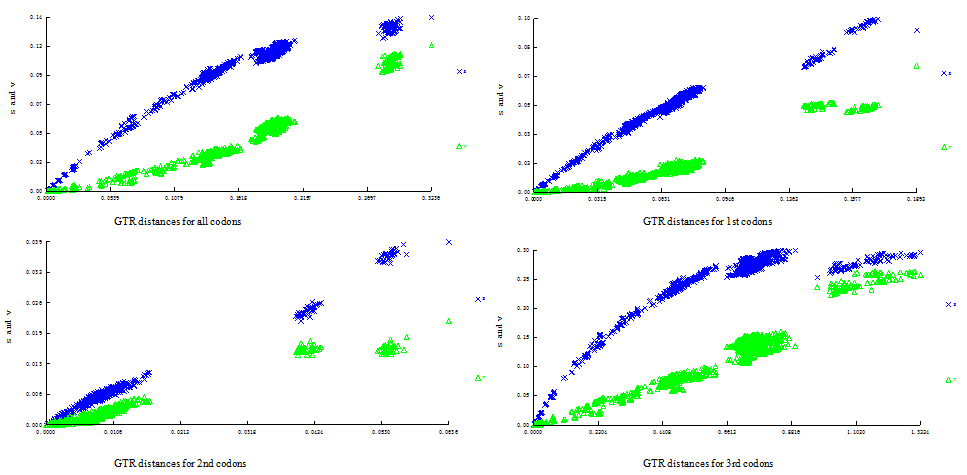

Supplement: Supplementary file 2 [file Image1.TIF]
